# The reference liver—CYP450 and UGT enzymes in healthy donor and metastatic livers: the impact of genotype

**DOI:** 10.1007/s43440-021-00337-w

**Published:** 2021-11-06

**Authors:** Mateusz Kurzawski, Sylwia Szeląg-Pieniek, Joanna Łapczuk-Romańska, Maciej Wrzesiński, Stefan Oswald, Marek Droździk

**Affiliations:** 1 Department of Experimental and Clinical Pharmacology, Pomeranian Medical University, Al. Powstańców Wlkp. 72, 70-111 Szczecin, Poland; 2 Department of General and Transplantation Surgery, Marie Curie Regional Hospital, Arkonska 4, 71-455 Szczecin, Poland; 3 Institute of Pharmacology and Toxicology, Rostock University Medical Center, 18051 Rostock, Germany

**Keywords:** Drug-metabolizing enzymes, Liver, Gene expression, Protein abundance

## Abstract

**Background:**

Hepatic enzymes involved in drug metabolism vary markedly in expression, abundance and activity, which affects individual susceptibility to drugs and toxicants. The present study aimed to compare mRNA expression and protein abundance of the most pharmacologically relevant drug-metabolizing enzymes in two main sources of the control liver samples that are used as the reference, i.e. organ donor livers and non-tumorous tissue from metastatic livers. An association analysis of the most common genetic variants with mRNA and protein levels was also performed.

**Methods:**

The CYP450 and UGT enzymes (CYP1A2, CYP2B6, CYP2C8, CYP2C9, CYP2C19, CYP2D6, CYP2E1, CYP3A4, CYP3A5, UGT1A1, UGT1A3, UGT2B7 and UGT2B15) were analyzed for mRNA (qPCR) and protein abundance (LC–MS/MS) in healthy donors (*n* = 11) and metastatic (*n* = 13) livers. Genotyping was performed by means of TaqMan assays and pyrosequencing.

**Results:**

Significantly higher protein abundance in the metastatic livers was observed in case of CYP2C9, CYP2D6, and UGT2B7, and for UGT1A3 the difference was only significant at mRNA level. For all the enzymes except CYP2E1 some significant correlation between mRNA and protein content was observed, and for UGT1A1 an inverse correlation with age was noted. CYP2C19, CYP3A5 and CYP2D6 were significantly affected by genotype.

**Conclusion:**

The selection of a control group for the study on drug-metabolizing enzymes (e.g. in pathological states) may possibly affect its conclusions on differences in mRNA and protein content. Genotyping for common functional variants of CYP450 enzymes should be performed in all studies on drug-metabolizing enzymes.

**Supplementary Information:**

The online version contains supplementary material available at 10.1007/s43440-021-00337-w.

## Introduction

Most of the drugs, as well as other xenobiotics, undergo biotransformation in the body, which has a significant impact on their pharmacokinetics and, in consequence, their actions and biological effects. Although drug metabolism occurs in most tissues, liver is the major site of biotransformation. Drug metabolism is divided into two phases: phase I reactions usually increase polarity of compounds and might possibly be followed by the second phase—conjugation, which generally serve as a final detoxifying and inactivating step [1]. The cytochromes P450 (CYPs) constitute the major enzyme family capable of catalyzing oxidative biotransformation of most drugs, as well as many endogenous substrates. Among over fifty human CYPs, those of particular relevance for clinical pharmacology include hepatic CYP1A2, CYP2B6, CYP2C8, CYP2C9, CYP2C19, CYP2D6, CYP2E1, CYP3A4, and CYP3A5 [2]. As for phase II of drug metabolism, conjugation with glucuronic acid is the most common pathway of drug biotransformation, and the UDP-glucuronyltransferases from the UGT1A and 2B subfamilies play a key role in terminating the biological actions and enhancing the renal elimination of non-polar drugs [3]. Hepatic enzymes involved in drug metabolism vary markedly in expression, abundance and activity, which affect individual susceptibility to drugs and toxicants. Apart from factors regulating gene expression and enzymatic activity, genetic variation has a large impact on the observed interindividual variability. In case of some cytochromes, i.e. CYP2D6, CYP3A5 or CYP2C19, common genetic variants may even result in complete deficiency of enzymatic activity in a significant number of patients [2].

A number of papers have been published, focusing on altered levels of drug-metabolizing enzymes (DMEs) in various liver pathologies, i.e. viral hepatitis, alcoholic, immunological and cholestatic liver diseases, inflammation, non-alcoholic fatty liver disease and hepatic cancer [4–9]. Despite the quantification method used (mRNA expression, protein abundance or enzymatic activity), final conclusions were drawn from comparisons of pathological liver specimens and controls (considered as healthy) liver tissues. There are two main sources of the control liver samples used as reference livers in published studies, i.e. donor livers [5, 7, 9] and non-tumorous tissue from metastatic livers [4, 8], and in some of the studies combined control groups were used [6]. As it is not clear if liver DMEs are significantly affected by metastatic cancer or if they are altered in brain-dead organ donors (but both groups could represent the same functional liver state in a routine clinical screening), the present study aimed, for the first time, to characterize mRNA expression and protein abundance of the most pharmacologically relevant DMEs in the two most frequently used types of reference liver tissues. Secondly, even though it is clear that genetic factors have significant impact on individual variation and function of some DMEs, genetic analysis is not always included in liver studies [5, 10, 11]. Hence, association of the most common functional genetic variants of genes encoding the studied DMEs with mRNA and protein levels was also investigated in the current paper.

The current study is complementary to the previously published paper, which demonstrates differences of drug transporter expression and protein abundance (ABC: P-gp, MRP1, MRP2, MRP3, MRP4, BCRP, BSEP and SLC: NTCP, MCT1, OCT1, OCT3, OAT2, OATP1B1, OATP1B3, OATP2B1) in metastatic and donor livers [12]. The protein abundance of NTCP was significantly higher, whereas of P-gp significantly lower in non-tumorous tissues from metastatic livers.

## Materials and methods

### Liver samples

Tissues were sampled from two groups of individuals: (1) organ donors (*n* = 11), and (2) patients undergoing surgical resection of liver metastases (*n* = 13). All the subjects were Polish of European descent. The deceased organ donors, died from intracranial bleeding or head injury, were free from chronic diseases. A small sample of a donor liver was taken from the explanted liver, just after resection. The samples from metastatic patients, i.e. non-tumorous liver tissue (which was confirmed by histological examination), located at least 5 cm aside from metastatic tumors, were collected during resection of metastatic colon adenocarcinoma from patients, who were free from chronic diseases. The specimens were immediately snap frozen in liquid nitrogen for protein analysis or put in RNAlater (Applied Biosystems, USA) for RNA analysis, and subsequently stored in − 80 °C until further processing. Clinical characteristics of the study participants is given in Table 1 (most of the samples were previously used [12]). The collected liver tissues did not show any signs of inflammation or necrosis as confirmed by histological examination. The study was carried out in accordance with the Declaration of Helsinki and the study protocol was approved by the local Bioethics Committee at the Pomeranian Medical University.

**Table 1:** Clinical characteristics of the study groups (mean ± SD, range)

	Organ donors (*n* = 11)	Patients with metastatic liver (*n* = 13)	*p*^a^
Age (years)	46.2 ± 12.6 (19–60)	64.1 ± 8.3 (53–77)	*3* × *10*^*–4*^
Sex (males/females)	8/3	9/4	*0.792*
Total bilirubin [< 1.23 mg/dL]	0.70 ± 0.69 (0.1–2.15)	0.55 ± 0.26 (0.20–1.06)	*0.710*
AST [5–40 IU/L]	33 ± 10 (12–45)	37 ± 19 (11–65)	*0.756*
ALT [5–40 IU/L]	30 ± 20 (13–89)	29 ± 18 (10–55)	*0.797*
Serum total protein [6.0–8.0 g/dL]	5.2 ± 1.0 (3.3–6.4)	6.8 ± 0.7 (5.2–7.5)	*0.002*
Serum albumin [3.5–5.5 g/dL]	2.8 ± 0.7 (1.5–3.6)	3.8 ± 0.4 (3.4–4.4)	*0.003*
INR [0.8–1.2]	1.23 ± 0.13 (1.0–1.4)	1.13 ± 0.21 (0.9–1.7)	*0.051*
Creatinine [0.6–1.3 mg/dL]	1.13 ± 0.54 (0.6–1.84)	0.90 ± 0.23 (0.54–1.34)	*0.477*
Administered drugs	Dopamine, dobutamine, epinephrine, lidocaine, sodium nitroprusside, vasopressin, cephalosporines, gentamycin, vancomycin, clindamycin, mannitol	Sevoflurane, propofol, rocuronium, fentanyl, oxycodone, midazolam	

^a^*p* value calculated by means of Mann–Whitney *U* test, except for sex ratio (Chi^2^ test with Yate’s correction); [ ]—normal range

### mRNA quantification

Total RNA was isolated from 20 mg of tissue specimen by means of mirVana kit (ThermoFisher Scientific, USA—donors’ group) or Direct-zol RNA MiniPrep kit (Zymo Research, USA). The sample storage time varied from 1 to 42 months. RNA quality was verified by RIN (RNA integrity values) with 2100 Bioanalyzer (Agilent, USA) prior to further analysis. Reverse transcription was performed using SuperScript VILO Master Mix (Thermo Fisher Scientific, USA), using 500 ng of total RNA for 20 µL of reaction volume, according to a protocol from the supplier. Relative expression of transporter gene expression was determined by means of real-time PCR, using ViiA 7 Real-Time PCR System (Life Technologies, USA), TaqMan Fast Advanced Master Mix and pre-validated TaqMan assays (Thermo Fisher Scientific, USA). Threshold values were equal for all the genes, and *C*_T_ values were used to calculate relative transcript concentrations (Δ*C*_T_ method). Mean *C*_T_ value of five reference genes: *GAPDH, PPIA, HMBS, RPLP0*, and *RPS9* was used as a reference for quantification of relative expression of the investigated DMEs genes. Details of TaqMan assays are provided in Supplementary Table 1.

### Genotyping

Genomic DNA was extracted from liver samples by means of Tissue DNA Purification Kit (EURx, Poland). All samples were genotyped for the presence of the most relevant variants, associated with activity of the studied enzymes (polymorphisms with previously established functional consequences with minor allele frequency MAF > 0.05 in the studied population). Genetic analysis was performed with the use of ViiA 7 Real-Time PCR System, pre-validated Drug Metabolism TaqMan Genotyping Assays and TaqMan GTXpress Master Mix (Thermo Fisher Scientific, USA) in 6 µL reaction volume. Fluorescence data was captured after 40 cycles of PCR. Details of the investigated single nucleotide polymorphisms (SNPs) and assays are given in Supplementary Table 2. The UGT1A1*28 allele status was determined by means of pyrosequencing, using PyroMark Q48 Autoprep instrument (Qiagen, USA) and primer sequences previously described by Sukasem et al. [13].

### Protein quantification by LC–MS/MS

Protein quantification of CYP450 and UGT enzymes (CYP1A2, CYP2B6, CYP2C8, CYP2C9, CYP2C19, CYP2D6, CYP2E1, CYP3A4, CYP3A5, UGT1A1, UGT1A3, UGT2B7 and UGT2B15) was performed by means of mass spectrometry-based targeted proteomics using a validated LC–MS/MS method, as recently described [14, 15]. In brief, about 40 mg of pulverized tissue was added to 1 mL lysis buffer (0.2% SDS, 5 mM EDTA) containing 5 µL/mL Protease Inhibitor Cocktail (ProteoExtract-Native Membrane Extraction Kit; Merck, Darmstadt, Germany), and manually homogenized using a Dounce homogenizer, and subsequently incubated for 30 min at 4 °C. After determination of the protein concentration (Pierce BCA Protein Assay Kit; Thermo Fisher Scientific, Germany), a volume corresponding to 100 µg protein was subjected to the established method of filter-aided sample preparation. The obtained data were normalized to the respective mass of tissue lysate used in the tryptic digest. LC–MS/MS analyses were conducted on API4000 triple quadrupole mass spectrometer (AB Sciex, Foster City, CA, USA) coupled to a Shimadzu LC (SLC-10A VP) system (Shimadzu, USA) and an HTS PAL LEAP autosampler (LEAP Technologies, USA). The details of the procedure, used peptides and mass transitions were previously described elsewhere [15].

### Statistical methods

Distribution of the analyzed variables (i.e. mRNA and protein abundance) was tested for normality using Shapiro–Wilk test. Due to a significant deviation from normal distribution, differences between study groups were further evaluated by means of non-parametric Mann–Whitney *U* test. The correlations were measured with Spearman’s rank (*R*^2^) correlation coefficient. All calculations were performed using Statistica 13.1 software package (TIBCO Software Inc., Palo Alto, CA, USA).

## Results

**Fig. 1:**
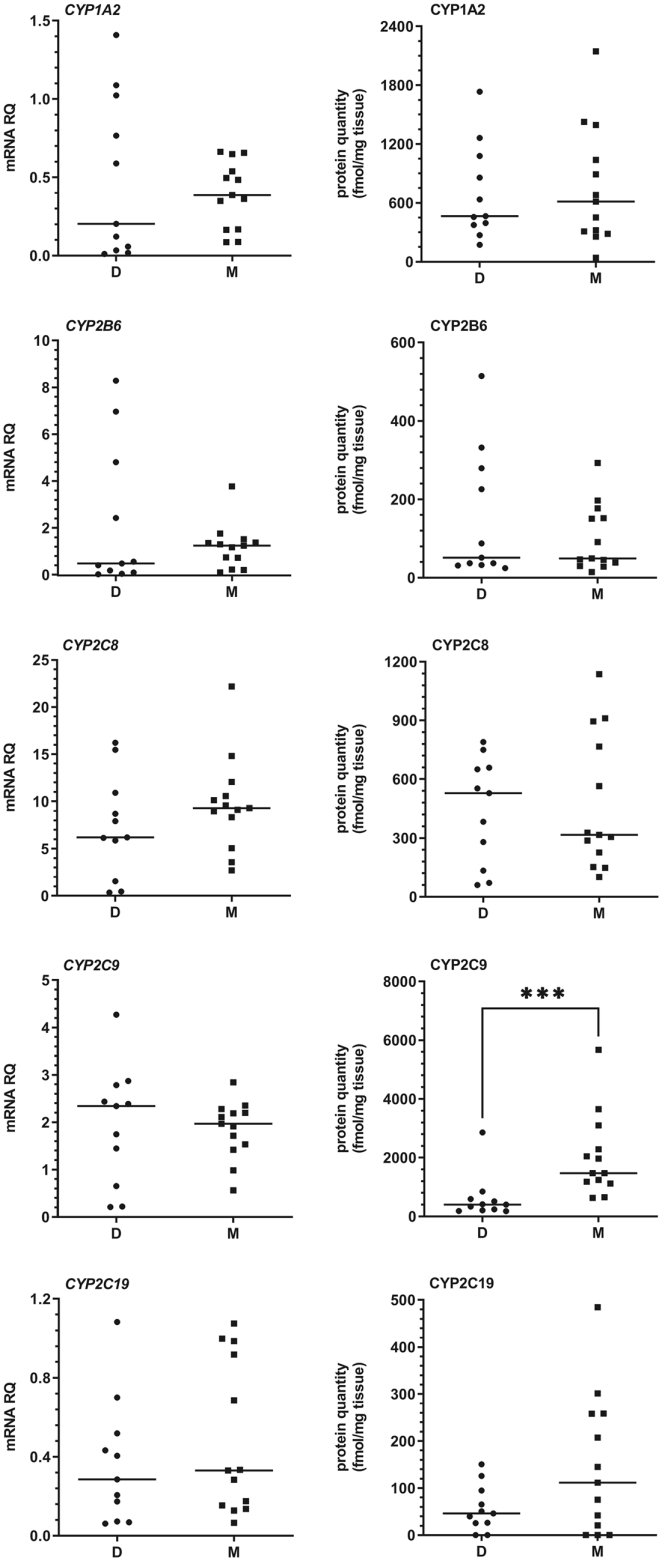
The mRNA and protein abundance of CYP1A2, CYP2B6, CYP2C8, CYP2C9, and CY2C19 in livers from organ donors (*D*) and patients undergoing metastatic tumor resection (*M*). Horizontal bars represent median values for each group. Values for mRNA are shown as relative quantity in relation to mean expression of five housekeeping genes (*GAPDH, PPIA, HMBS, RPLP0*, and *RPS9)*. Significant *p* values are marked with asterisks ***p < 0.001)

**Fig. 2:**
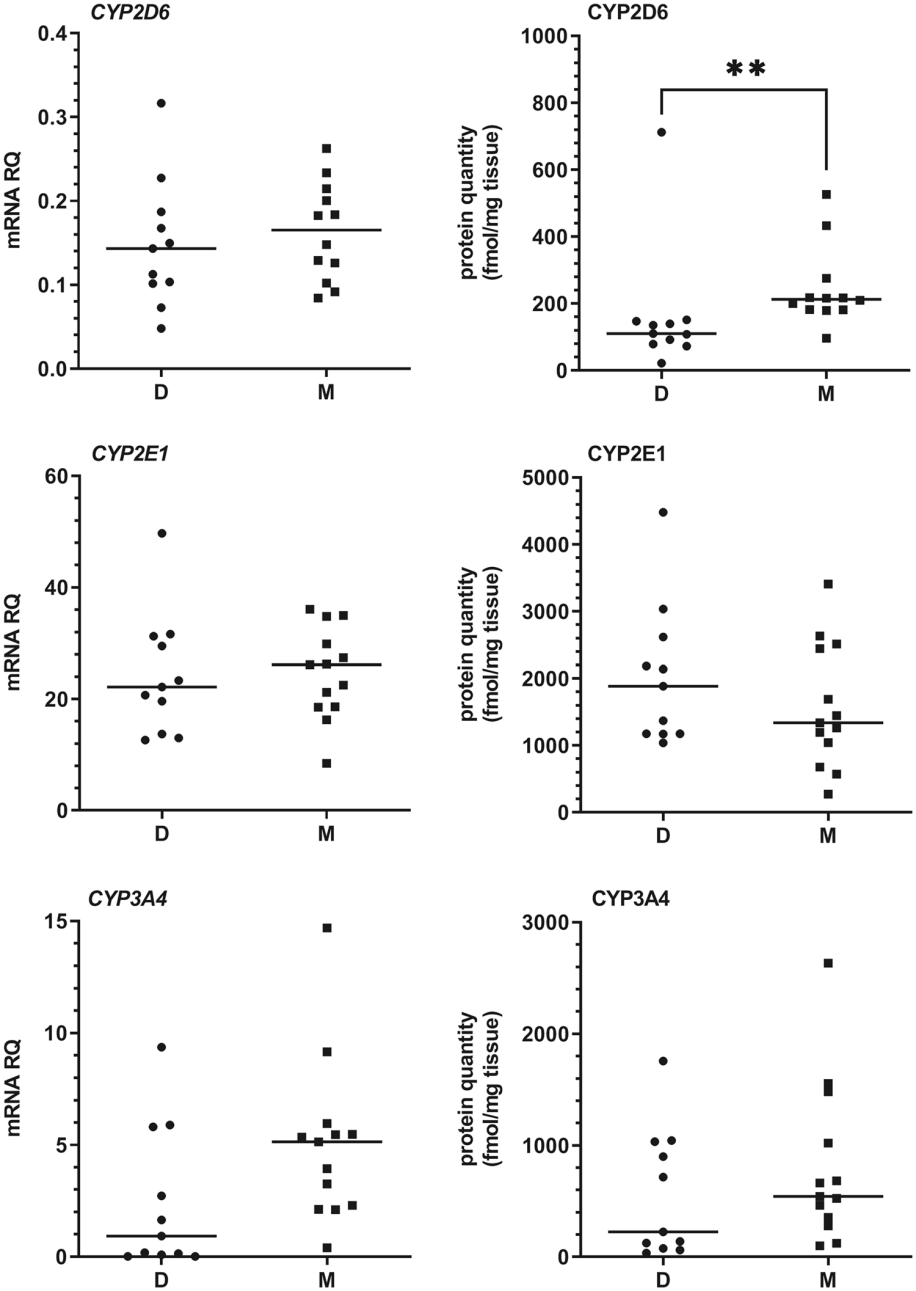
The mRNA and protein abundance of CYP2D6, CYP2E1, and CYP3A4 in livers from organ donors (*D*) and patients undergoing metastatic tumor resection (*M*). Horizontal bars represent median values for each group. Values for mRNA are shown as relative quantity in relation to mean expression of five housekeeping genes (*GAPDH, PPIA, HMBS, RPLP0*, and *RPS9)*. Significant *p* values are marked with asterisks (**p < 0.01)

Among investigated CYP450 enzymes, CYP2E1, CYP3A4, CYP2C9, CYP2C8, and CYP1A2 proteins were present at the highest concentrations in the liver tissues in both study groups (i.e. organ donors and metastatic colon cancer patients), while CYP2D6, CYP2B6 and CYP2C19 protein content were distinctly lower. Total abundance of all the analyzed CYP proteins was about 25% higher in the metastatic livers. Although the rank order of particular CYPs proteins was different in the study groups [decreasing abundance order—mean values: CYP2E1 (43%) > CYP1A2 (15%) > CYP2C9 (13%) > CYP3A4 (12%) > CYP2C8 (9%) > CYP2D6 (3%) > CYP2B6 (3%) > CYP2C19 (1%) in donors, and CYP2C9 (32%) > CYP2E1 (25%) > CYP3A4 (13%) > CYP1A2 (12%) > CYP2C8 (8%) > CYP2D6 (4%) > CYP2C19 (2%) > CYP2B6 (2%) in metastatic patients], significant differences were only observed in case of CYP2C9 (*U* = 12, *N*_1_ = 11, *N*_2_ = 13, *p* = 0.0002) and CYP2D6 (*U* = 29, *N*_1_ = 11, *N*_2_ = 13, *p* = 0.0129) (higher mean and median values in the metastatic livers, Figs. 1 and 2). Quantitative analysis of mRNA did not reveal any significant differences in the expression of CYP genes between the study groups. Nevertheless, a significant correlation between mRNA and protein content for most investigated CYPs, except for CYP2E1 and CYP2C9 (combined analysis of all study samples) was observed (Table 2).

**Table 2:** Correlation between RNA and protein abundance of CYP450 and UGT enzymes in human liver samples

mRNA vs. protein	*D* *n* = 11	*M* *n* = 13	All samples *n* = 24
CYP1A2	0.845**	0.775**	0.744***
CYP2B6	0.773**	0.593	0.790***
CYP2C8	0.909***	0.786**	0.790***
CYP2C9	0.764**	0.802***	0.381
CY2C19	0.601	0.884***	0.763***
CYP2D6^a^	0.664*	0.525	0.493*
CYP2E1	− 0.036	0.258	0.138
CYP3A4	0.845**	0.780**	0.851***
CYP3A5	0.691*	0.676*	0.744***
UGT1A1	0.609*	0.626*	0.722***
UGT1A3	0.236	0.527	0.430*
UGT2B7	0.782**	0.670*	0.799***
UGT2B15	0.355	0.813***	0.492*

Spearman coefficient values are given

*M* non-tumorous liver samples from patients with metastatic colon cancer, *D* liver samples from organ donors

*p < 0.05

**p < 0.01

***p < 0.001

^a^Patient deficient for CYP2D6 (*4/*4 genotype) was excluded from analysis

As for CYP3A5, it is known that a common genetic variant (CYP3A5*3) causes alternative splicing and protein truncation, finally resulting in complete absence of CYP3A5 activity [16]. Since only three individuals inherited a functional CYP3A5*1 allele (all in the metastatic group), CYP3A5 was excluded from the comparison and subsequently analyzed only in the context of genotype impact on mRNA and protein quantity.

**Fig. 3:**
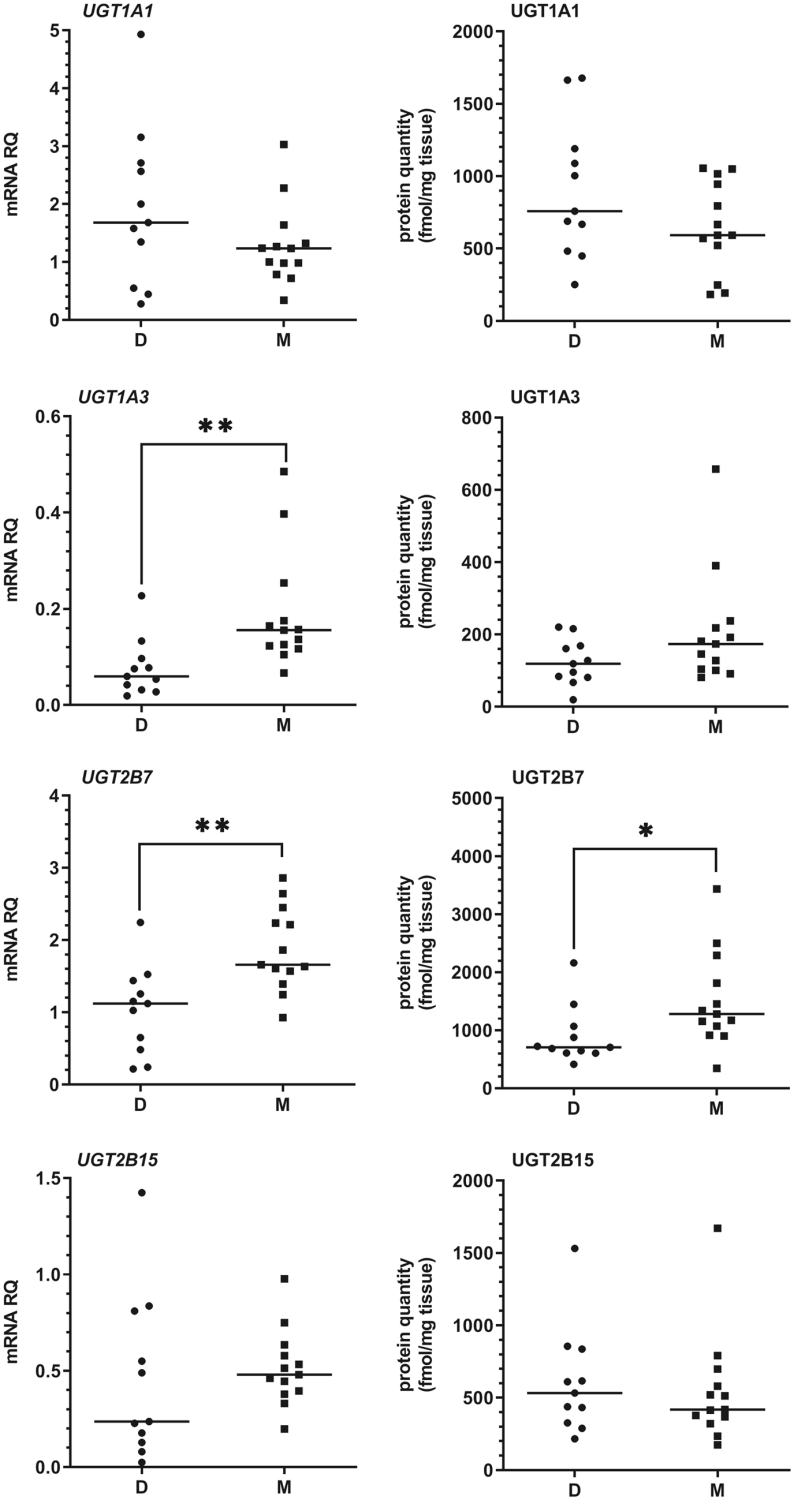
The mRNA and protein abundance of the most pharmacologically relevant UGT enzymes (UGT1A1, UGT1A3, UGT2B7, and UGT2B15) in livers from organ donors (*D*) and patients undergoing metastatic tumor resection (*M*). Horizontal bars represent median values for each group. Values for mRNA are shown as relative quantity in relation to mean expression of five housekeeping genes (*GAPDH, PPIA, HMBS, RPLP0*, and *RPS9*). Significant *p* values are marked with asterisks (*p < 0.05; **p < 0.01)

As for UGT enzymes, UGT1A1 and UGT2B7 demonstrated the highest abundances in both study groups, followed by UGT2B15, and a minor content of UGT1A3. There was a significantly increased abundance of hepatic UGT2B7 (*U* = 29, *N*_1_ = 11, *N*_2_ = 13, *p* = 0.0129) in metastatic patients when compared to the organ donors, which was also observed in mRNA analysis (*U* = 21, *N*_1_ = 11, *N*_2_ = 13, *p* = 0.0025) (Fig. 3). A significant correlation between mRNA and protein content was noted in the case of all analyzed UGTs. Details of mRNA and protein quantitative analysis is presented in Figs. 1, 2, 3 and Supplementary Tables 3 and 4.

**Fig. 4:**
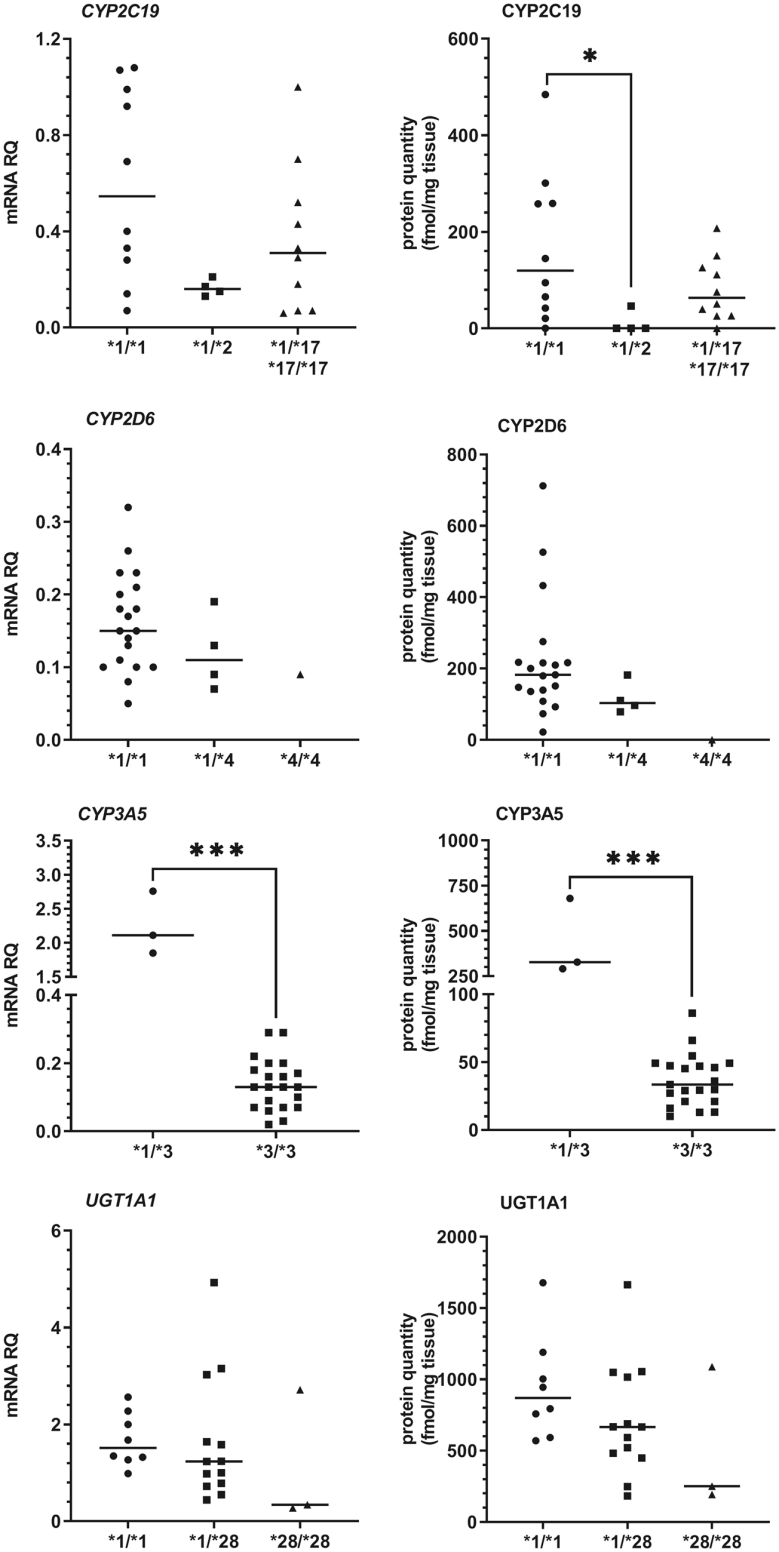
The impact of genotype on mRNA and protein abundance of drug-metabolizing enzymes (CYP2C19, CYP3A5, CYP2D6, and UGT1A1) in all study participants (*n* = 24). Horizontal bars represent median values for each group. Values for mRNA are shown as relative quantity in relation to mean expression of five housekeeping genes (*GAPDH, PPIA, HMBS, RPLP0*, and *RPS9*). Significant *p* values are marked with asterisks (*p < 0.05; ***p < 0.001)

Apart from quantitative comparison of two reference liver tissues, an analysis of common functional genetic variants within DMEs genes was performed in all 24 study samples. Among the studied variants, a significant impact of CYP2C19*2 and CYP3A5*3 alleles was observed. The carriers of CYP2C19*2 variant were characterized by decreased mRNA levels and protein abundance of CYP2C19 enzyme, and the genotype-dependent difference was significant at protein level (H = 6.4, *N*_1_ = 10, *N*_2_ = 4, *N*_3_ = 10, *p* = 0.0417), as three of four CYP2C19*1/*2 individuals ware characterized by a protein abundance below level of detection of the applied method of quantification (Fig. 4). Similarly, mRNA and protein content in CYP3A5*3 (*U* = 0, *N*_1_ = 3, *N*_2_ = 21, *p* = 0.001) homozygotes were markedly lower compared than in carriers of wild-type CYP3A5*1 allele. In case of CYP2D6, carriers of the non-functional CYP2D6*4 allele were not significantly affected by the polymorphism (*p* = 0.075 for protein content). However, an individual with CYP2D6*4/*4 homozygous genotype represented the only case with undetectable CYP2D6 protein. Contrary to the aforementioned variants, resulting in splicing defect and processing of non-functional protein, none of the other investigated genetic polymorphisms (missense SNPs or located in promoters/introns) showed any association with gene expression or protein abundance of the investigated DMEs (Supplementary Tables 5 and 6). Although UGT1A1*28 association with decreased gene expression and Gilbert syndrome is well documented, and could be observed in the current study (Fig. 4), the inter-genotype differences were not significant, possibly due to a relatively small number of samples and considerable interindividual variability.

**Fig. 5:**
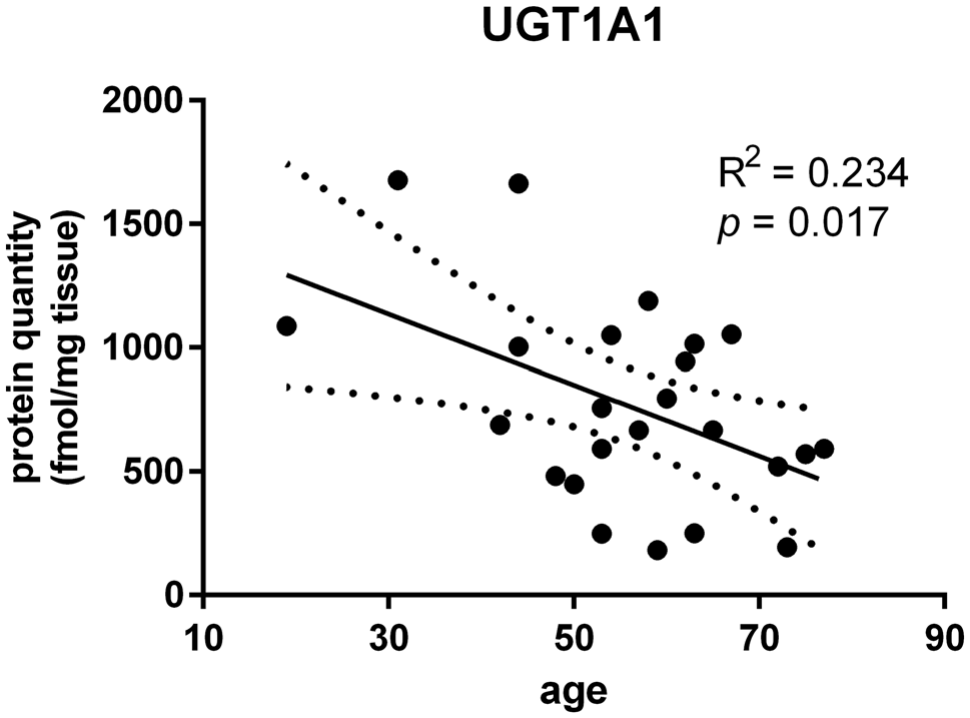
Linear regression analysis of the relation between patient’s age and UGT1A1 protein abundance in the human liver

Finally, we have analyzed mRNA relative quantity and protein abundance of DMEs in relation to a patient’s age and sex. A significant impact of age on UGT1A1 protein abundance was observed, with decreasing UGT1A1 liver levels in elderly subjects (*R*^2^ = 0.234, *p* = 0.017, Fig. 5). No other age-dependent associations were shown. Similarly, we did not observe any significant differences between men and women, neither in mRNA quantity, nor in protein abundance (Supplementary Tables 7 and 8).

## Discussion

In the current study, we compared for the first time the expression of DMEs genes of two sets of liver samples, i.e. non-tumoral liver tissue from patients undergoing resection of colon cancer metastases and liver samples from organ donors (liver donors), that are widely used as reference samples, presumably healthy livers [4–9]. Gene expression was measured both at mRNA and protein levels. Several significant differences between the aforementioned types of liver tissues were identified.

As for CYP450 enzymes, significantly higher protein content of hepatic CYP2C9 and CYP2D6 was noted in metastatic patients, when compared with organ donors. CYP2C9 is mainly regulated by nuclear receptors: the constitutive androstane receptor (CAR) and pregnane X receptor (PXR), that control transcription of many CYP450 genes [17]. It was demonstrated that pro-inflammatory cytokines (e.g., IL-1, IL-6, and TNF-α) downregulate hepatic expression of CYP1, CYP2, CYP3, and CYP4 families. Since the inflammatory process is usually present in cancer patients, both in the tumor microenvironment and systemically, one could rather expect decreased CYP2C9 expression in metastatic patients [18]. However, it was shown that CYP2C9 was downregulated by IL-6 and TGF, but not significantly affected by TNF, IFN, or IL-1 in human hepatocytes, contrary to CYP3A4 and CYP2C8, which were downregulated by all aforementioned cytokines. Those data suggest that cytokine-related regulatory process may be more complex and gene-specific.

Although genetic polymorphism is the main contributor to the interindividual variability of CYP2D6 activity, it does not explain all the observed differences. It was previously documented the urinary metabolic ratio of dextromethorphan or debrisoquine (CYP2D6 substrates) varied greatly among individuals carrying the wild-type *CYP2D6* alleles [19]. However, contrary to most of the DMEs of the CYP450 system, it is not regulated by CAR nor PXR. Potential transcriptional regulation may involve hepatocyte nuclear factor 4a (HNF4a), small heterodimer partner (SHP) and hepatic concentration of retinoids [19], but the current knowledge is still very limited and does not allow to define mechanisms underlying the observed differences between the groups in the current study.

Although the rank of CYP450 enzymes arranged based on decreasing abundance differed between the two groups, four enzymes detected at the highest quantity were the same, i.e. CYP2E1, CYP1A2, CYP2C9 and CYP3A4 (all over 10% of the analyzed CYP450 fraction in both groups). This is in concordance with data presented in the paper of Prasad et al. for control tissue from organ donors [5], except the high content of CYP2A6 enzyme reported by those authors. As CYP2A6 is usually not considered as pharmacologically relevant, it was not included in the current study. Similarly, CYP3A, CYP2C (without discrimination between individual members) and CYPA2 were pointed as major hepatic fraction of CYP450 “pie” in the paper by Michaels and Wang [11]. Those authors reported a high content of CYP4F protein, but again, it was not included in the current study, which was focused on pharmacologically relevant enzymes. As for the CYP2E1, lower protein content was reported in the aforementioned paper (6.6%), but it might be related with the fact that CYP2E1 is more prone to degradation and thus sensitive to prolongation of sample harvesting and processing time, as it was discussed before [20].

As for UGTs, the only significant difference at the protein level was noticed in case of UGT2B7. Contrary to members of the UGT1A family, encoded by a single locus et 2q37 (sharing common exons), UGT2B7 is located at chromosome 4 and its expression seems not to be regulated by xenobiotics via CAR/PXR/AhR, but is rather controlled by toxic bile acids (mainly lithocholic acid, LCA) via FXR [21]. LCA is a product of conversion of chenodeoxycholic acid by intestinal bacteria. As both increased LCA level and altered gut microbiota are risk factors for colorectal cancer, this can be a potential reason of higher *UGT2B7* expression in the livers from metastatic patients compared to deceased organ donors [22]. However, bile acid concentrations should be measured to verify that hypothesis.

The two patient groups differed in relation to drugs administered before liver sample collection (Table 1). Some of those drugs are extensively metabolized in liver in reactions catalyzed by the enzymes investigated in the current study, e.g. midazolam (CYP3A4 and UGT1A4) or oxycodone (CYP3A4, CYP2D6) [23]. However, none of the drugs is a significant enzymatic inducer or inhibitor, so the observed differences in mRNA/protein content of drug-metabolizing enzymes are unlikely related to pharmacological treatment.

It is generally accepted that genetic variation is one of the major determinants of interindividual and ethnic differences in activity of DMEs in humans [24]. Despite that fact, many studies related to hepatic expression of DMEs genes, protein quantification or enzymatic activity did not include genetic analysis. In the current paper, we observed a significant impact of *CYP2C19* genotype on protein abundance, with significant decrease of protein abundance in patients heterozygous for *2 allele. The rs4244285 polymorphism, defining the CYP2C19*2 allele, creates an exon 5 aberrant splice site, altering the reading frame of the mRNA and leading to a premature stop codon and formation of a non-functional protein [25]. A more recent study has demonstrated, that another SNP (rs12769205 in intron 2, also found in all CYP2C19*2 alleles) leads to intron 2 retention, and may produce complete loss of CYP2C19 protein [26]. Since allele *2 is quite common, especially in Asian populations, genotyping should be definitely included in the studies related to *CYP2C19* variable expression in humans. Contrary, we did not confirm an association of CYP2C19*17 (promoter variant), previously pointed as a gain-of-function allele, with increased transcriptional activity, which does not support its major significance in relation to CYP2C19 abundance in the liver [27].

Another important genetic factor that should be definitely included in DME studies is the common *3 variant of *CYP3A5* gene. CYP3A5*3, the main determinant of that CYP expression levels in most populations, is characterized by the presence of rs776746 SNP in intron 3, which leads to alternative splicing, the insertion of an intronic sequence into mRNA, protein truncation and complete loss of activity [16]. The frequency of CYP3A5*3 allele is very high, reaching over 90% in Europeans (www.pharmgkb.org). The mRNA assay used in the current study could detect both wild-type (*1) and *3 transcripts, due to location in exons 2–3. The peptide used for protein quantitation by MS is located in exon 8, so should not detect signal in case of CYP3A5*3. However, it was presented that the chimeric CYP3A proteins, characterized by CYP3A43 exon 1 joined to distinct sets of CYP3A4 or CYP3A5 exons are present in the human liver [28], which may explain weak signal detected by our method in samples homozygous for CYP3A5*3 allele. In one of previous studies we have reported that CYP3A5 is present at very low levels in the human liver, and constituted only about 1% of liver CYP content [15]. The current study revealed that CYP3A5 protein abundance may be much higher (nearly comparable with that of CYP3A4), but this is limited to so-called CYP3A5 “expressers”, i.e. individuals possessing at last one functional allele of the *CYP3A5* gene Fig. 4). Similarly, results of other studies referring to CYP3A5 could possibly not reflect the real status of the enzyme as the patients were not genotyped for CYP3A5*3 allele [5, 10, 11].

The hepatic CYP2D6 protein abundance varies dramatically from person to person mainly due to its genetic polymorphism [29]. In case of CYP2D6, the presence of a single *4 allele was not significantly associated with decreased levels of CYP2D6 mRNA or protein, which could be due to relatively small number of subjects in the current study. For the same reason, we did not detect any copy number variation of *CYP2D6* gene among the study participants, so the effect of gene deletion/multiplication could not be verified. However, an individual with *4/*4 homozygous genotype was the only case with undetectable CYP2D6 protein level, which supports the necessity of genotyping subjects involved in CYP450 expression/activity studies.

Additional analyses of mRNA and protein content were performed in relation to a patient’s age and sex. We were not able to confirm previously reported differences in CYP3A4, which is considered is to be female predominant [10]. We have observed an inverse correlation between patients age and UGT1A1 protein content, but it is not in concordance with the previous studies on activity of UGT1A enzymes [30, 31]. However, due to limited sample size and potential influence of other factors, the results of these analyses should be treated with a special caution.

Although some significant observations were reported in the current study, it should be clearly stated that there are some important limitations. First of all, only limited number of samples was included, which does not allow detection of some minor differences. It is known that some diet components may inhibit enzymatic activity (e.g. grapefruit juice in case of CYP3A4), while nicotine is an inducer of CYP1A2, but no data on cigarette smoking or diet was available in the current study. Additionally, rare functional genetic variants were not analyzed in the current study, so their presence in the study subjects cannot be excluded.

It should also be emphasized that not the mRNA content or protein abundance finally determine drug metabolism in an individual patient, but the enzymatic activity. Even if there is a significant correlation between mRNA/protein content and activity measurements in case of many DMEs, the correlation is not full [32]. Secondary, not all the DMEs are regulated at transcriptional level, e.g. the induction of CYP2E1 seems to be regulated posttranslationally by protection against the rapid degradation of protein in the liver [33]. In relation to pharmacogenetic issues, not all the functional polymorphisms of DME genes affect gene expression or protein abundance, what has been also observed in the current study. It was previously documented that some missense variants (like in case of CYP2C9 *2 and *3 or CYP2B6*6 alleles) do not influence protein content nor stability, but modify enzymatic activity and substrate specificity [34, 35]. Thus, if possible, phenotype should be assessed in drug metabolism studies, as the best determinant of drug response.

Finally, the current study revealed differences in mRNA and protein levels of DMEs between the donor livers and the non-tumorous tissue from metastatic livers. Our results show, that in case of some enzymes, i.e. CYP2C9, CYP2D6 and UGT2B7, the selection of a control group for the study is important and may affect certain conclusions. On the other hand, genetic variation is an important determinant of some enzymes’ gene expression and protein content, which is the most pronounced in case of CYP2C19, CYP2D6 and CYP3A5. The latter observation supports the necessity of genotype analysis incorporation into all studies on DMEs.

## Supplementary Information

Below is the link to the electronic supplementary material.

Supplementary file1 (DOCX 56 KB)
