# Supplementary material for: The reference liver—CYP450 and UGT enzymes in healthy donor and metastatic livers: the impact of genotype
Source: Pharmacol Rep. 2021 Nov 6;74(1):204–15. doi: 10.1007/s43440-021-00337-w (PMC8786777; doi:10.1007/s43440-021-00337-w)
Supplement: Supplementary file 1 — Supplementary file1 (DOCX 56 KB) [file 43440_2021_337_MOESM1_ESM.docx]

**Supplementary table 1.** List of assays used for mRNA quantification (|Thermo Fisher Scientific, USA).

| Gene symbol | Assay ID |
| --- | --- |
| *CYP1A2* | Hs00167927_m1 |
| *CYP2B6* | Hs03044631_m1 |
| *CYP2C8* | Hs04183483_g1 |
| *CYP2C9* | Hs02383631_s1 |
| *CYP2C19* | Hs00426380_m1 |
| *CYP2D6* | Hs00164385_m1 |
| *CYP2E1* | Hs00559367_m1 |
| *CYP3A4* | Hs00604506_m1 |
| *CYP3A5* | Hs01070905_m1 |
| *UGT1A1* | Hs02511055_s1 |
| *UGT1A3* | Hs04194492_g1 |
| *UGT2B7* | Hs00426592_m1 |
| *UGT2B15* | Hs00870076_s1 |
| *GAPDH* | Hs99999905_m1 |
| *PPIA* | Hs99999904_m1 |
| *HMBS* | Hs00609297_m1 |
| *RPLP0* | Hs99999902_m1 |
| *RPS9* | Hs02339424_g1 |

**Supplementary table 2.** List of assays used for genotyping (Thermo Fisher Scientific, USA).

| Gene variant | SNP id | Assay ID | Type of polymorphism |
| --- | --- | --- | --- |
| *CYP2B6*6* | rs3745274 | C___7817765_60 | missense |
| *CYP2C8*3* | rs11572080 | C__25625794_10 | missense |
| *CYP2C8*4* | rs1058930 | C__25761568_20 | missense |
| *CYP2C9*2* | rs1799853 | C__25625805_10 | missense |
| *CYP2C9*3* | rs1057910 | C__27104892_10 | missense |
| *CYP2C19*2* | rs12769205 | C__25744790_10 | splicing defect |
| *CYP2C19*3* | rs4986893 | C__27861809_10 | missense |
| *CYP2C19*17* | rs12248560 | C____469857_10 | promoter |
| *CYP2D6*3* | rs35742686 | C__32407232_50 | frameshift |
| *CYP2D6*4* | rs3892097 | C__27102431_D0 | splicing defect |
| *CYP2D6*5* | gene deletion | Hs00010001_cn | deletion |
| *CYP2D6*1x2* | gene duplication | Hs00010001_cn | duplication |
| *CYP3A4*22* | rs35599367 | C__59013445_10 | intronic |
| *CYP3A5*3* | rs776746 | C__26201809_30 | splicing defect |
| *UGT2B7*2* | rs7662029 | C__30720663_20 | intronic, in linkage with missense |
| *UGT2B15*2* | rs1902023 | C__27028164_10 | missense |
|  |  |  |  |

**Supplementary Table 3**. Gene expression (relative mRNA quantity) of the CYPP450 and UGT enzymes in human liver samples

| **Gene symbol** | **median** | **CV%** | **min** | **max** | **mean** | **SD** | ***p*** |
| --- | --- | --- | --- | --- | --- | --- | --- |
| ***CYP1A2*** |  |  |  |  |  |  |  |
| D | 0.203 | 101 | 0.011 | 1.409 | 0.484 | 0.490 | 0.865 |
| M | 0.387 | 52 | 0.086 | 0.663 | 0.391 | 0.204 |  |
| ***CYPB6*** |  |  |  |  |  |  |  |
| D | 0.479 | 132 | 0.021 | 8.282 | 2.205 | 2.913 | 0.569 |
| M | 1.240 | 76 | 0.095 | 3.772 | 1.190 | 0.907 |  |
| ***CYP2C8*** |  |  |  |  |  |  |  |
| D | 6.207 | 71 | 0.351 | 16.221 | 7.261 | 5.172 | 0.228 |
| M | 9.313 | 49 | 2.714 | 22.182 | 9.724 | 4.797 |  |
| ***CYP2C9*** |  |  |  |  |  |  |  |
| D | 2.343 | 61 | 0.210 | 4.270 | 1.942 | 1.187 | 0.569 |
| M | 1.969 | 32 | 0.562 | 2.844 | 1.851 | 0.585 |  |
| ***CYP2C19*** |  |  |  |  |  |  |  |
| D | 0.285 | 82 | 0.061 | 1.082 | 0.364 | 0.300 | 0.649 |
| M | 0.331 | 77 | 0.066 | 1.075 | 0.482 | 0.373 |  |
| ***CYP2D6*** |  |  |  |  |  |  |  |
| D | 0.143 | 49 | 0.048 | 0.317 | 0.148 | 0.072 | 0.691 |
| M | 0.148 | 36 | 0.084 | 0.262 | 0.157 | 0.057 |  |
| ***CYP2E1*** |  |  |  |  |  |  |  |
| D | 22.162 | 43 | 12.652 | 49.690 | 24.284 | 10.396 | 0.691 |
| M | 26.120 | 32 | 8.443 | 36.072 | 24.694 | 7.856 |  |
| ***CYP3A4*** |  |  |  |  |  |  |  |
| D | 0.927 | 125 | 0.011 | 9.361 | 2.434 | 3.042 | 0.055 |
| M | 5.133 | 70 | 0.397 | 14.691 | 5.023 | 3.530 |  |
| ***CYP3A5*** |  |  |  |  |  |  |  |
| D | 0.091 | 67 | 0.024 | 0.294 | 0.124 | 0.083 | 0.106 |
| M | 0.163 | 142 | 0.068 | 2.762 | 0.635 | 0.900 |  |
| ***UGT1A1*** |  |  |  |  |  |  |  |
| D | 1.680 | 68 | 0.275 | 4.928 | 1.930 | 1.310 | 0.207 |
| M | 1.233 | 52 | 0.339 | 3.026 | 1.293 | 0.672 |  |
| ***UGT1A3*** |  |  |  |  |  |  |  |
| D | 0.060 | 75 | 0.019 | 0.227 | 0.077 | 0.057 | 0.001 |
| M | 0.156 | 62 | 0.067 | 0.485 | 0.189 | 0.117 |  |
| ***UGT2B7*** |  |  |  |  |  |  |  |
| D | 1.120 | 56 | 0.217 | 2.244 | 1.032 | 0.579 | 0.002 |
| M | 1.657 | 29 | 0.925 | 2.859 | 1.868 | 0.550 |  |
| ***UGT2B15*** |  |  |  |  |  |  |  |
| D | 0.237 | 90 | 0.025 | 1.425 | 0.453 | 0.408 | 0.361 |
| M | 0.480 | 37 | 0.198 | 0.977 | 0.513 | 0.189 |  |

Relative quantity of each transcript is presented, compared to mean expression of reference genes (ΔCT method); M – non-tumoral liver samples from patients with metastatic colon cancer; D – liver samples from organ donors; CV - coefficient of variation; p values calculated by means of Mann- Whitney U-test.

**Supplementary Table 4** Protein abundance of the of the P450s and UGTs in human liver samples

| **Protein** [fmol/mg] | **median** | **CV %** | **min** | **max** | **mean** | **SD** | **positive samples** | ***p*** |
| --- | --- | --- | --- | --- | --- | --- | --- | --- |
| **CYP1A2** |  |  |  |  |  |  |  |  |
| D | 465.22 | 66 | 172.63 | 1734.18 | 700.61 | 460.69 | 11/11 | 1.000 |
| M | 613.96 | 76 | 40.58 | 2144.41 | 758.28 | 579.72 | 13/13 |  |
| **CYPB6** |  |  |  |  |  |  |  |  |
| D | 51.02 | 105 | 24.59 | 514.52 | 150.17 | 157.06 | 11/11 | 0.776 |
| M | 49.17 | 81 | 14.28 | 292.68 | 100.94 | 82.01 | 13/13 |  |
| **CYP2C8** |  |  |  |  |  |  |  |  |
| D | 528.55 | 58 | 60.21 | 790.15 | 441.89 | 258.34 | 11/11 | 0.776 |
| M | 317.32 | 70 | 101.21 | 1136.74 | 472.59 | 330.54 | 13/13 |  |
| **CYP2C9** |  |  |  |  |  |  |  |  |
| D | 401.56 | 119 | 179.90 | 2858.21 | 617.56 | 734.33 | 11/11 | 2*10^-4^ |
| M | 1474.35 | 66 | 630.81 | 5676.82 | 2039.40 | 1351.99 | 13/13 |  |
| **CYP2C19** |  |  |  |  |  |  |  |  |
| D | 46.05 | 82 | 0.00 | 150.55 | 56.82 | 46.58 | 10/11 | 0.252 |
| M | 111.43 | 97 | 0.00 | 484.49 | 146.48 | 142.59 | 10/13 |  |
| **CYP2D6^a^** |  |  |  |  |  |  |  |  |
| D | 110.41 | 111 | 22.20 | 712.11 | 161.04 | 178.08 | 11/11 | 0.002 |
| M | 212.84 | 46 | 96.70 | 526.29 | 244.57 | 113.69 | 12/12 |  |
| **CYP2E1** |  |  |  |  |  |  |  |  |
| D | 1887.07 | 49 | 1038.94 | 4478.09 | 2024.72 | 1000.73 | 11/11 | 0.459 |
| M | 1340.22 | 56 | 272.07 | 3407.44 | 1576.63 | 886.84 | 13/13 |  |
| **CYP3A4** |  |  |  |  |  |  |  |  |
| D | 224.84 | 98 | 34.55 | 1756.92 | 555.20 | 545.15 | 11/11 | 0.392 |
| M | 541.99 | 86 | 99.21 | 2634.15 | 801.60 | 688.07 | 13/13 |  |
| **CYP3A5^b^** |  |  |  |  |  |  |  |  |
| D | 29.09 | 55 | 10.09 | 65.99 | 31.34 | 17.31 | 11/11 | 0.030 |
| M | 49.20 | 140 | 20.96 | 679.89 | 132.57 | 185.45 | 13/13 |  |
| **UGT1A1** |  |  |  |  |  |  |  |  |
| D | 757.70 | 50 | 251.59 | 1677.86 | 901.96 | 450.75 | 11/11 | 0.186 |
| M | 592.83 | 47 | 182.88 | 1054.66 | 648.42 | 301.67 | 13/13 |  |
| **UGT1A3** |  |  |  |  |  |  |  |  |
| D | 118.67 | 49 | 19.45 | 220.37 | 123.53 | 59.92 | 11/11 | 0.119 |
| M | 173.48 | 73 | 80.90 | 657.50 | 207.77 | 152.10 | 13/13 |  |
| **UGT2B7** |  |  |  |  |  |  |  |  |
| D | 705.00 | 53 | 412.15 | 2160.26 | 902.66 | 477.59 | 11/11 | 0.013 |
| M | 1278.58 | 52 | 344.22 | 3434.30 | 1512.17 | 783.79 | 13/13 |  |
| **UGT2B15** |  |  |  |  |  |  |  |  |
| D | 533.16 | 58 | 216.58 | 1531.99 | 607.87 | 352.51 | 11/11 | 0.459 |
| M | 418.49 | 67 | 174.36 | 1670.44 | 544.88 | 364.83 | 13/13 |  |

All results are given in fmol/mg of the analyzed tissue. M – non-tumoral liver samples from patients with metastatic colon cancer; D – liver samples from organ donors; CV - coefficient of variation; p values calculated by means of Mann-Whitney U-test; positive samples: number of samples with detectable level of the protein (>0.1 fmol/mg). ^a^Patient deficient for CYP2D6 (*4/*4 genotype) was excluded from analysis. ^b^Only three patients were CYP3A5 “expressers” – all in the M group.

**Supplementary table 5.** Analysis of an association between common genetic variants and mRNA expression of the studied drug-metabolizing enzymes.

|  | **n** | **Median (Q1-Q3)** | ***p*** |
| --- | --- | --- | --- |
| ***CYP2B6*** |  |  |  |
| *1/*1 | 9 | 1.24 (0.55-1.76) | 0.446 |
| *1/*6 | 15 | 0.73 (0.18-1.51) |  |
| ***CYP2C8*** |  |  |  |
| *1/*1 | 18 | 8.84 (5.88-10.92) | 0.768 |
| *1/*3 | 5 | 8.34 (5.06-9.31) |  |
| *3/*4 | 1 | 10.12 |  |
| ***CYP2C9*** |  |  |  |
| *1/*1 | 13 | 2.28 (1.75-2.39) | 0.457 |
| *1/*2 | 5 | 1.72 (0.98-1.91) |  |
| *1/*3 | 5 | 1.97 (1.45-2.11) |  |
| *2/*3 | 1 | 1.53 |  |
| ***CYP2C19*** |  |  |  |
| *1/*1 | 10 | 0.55 (0.28-0.99) | 0.158 |
| *1/*2 | 4 | 0.16 (0.14-0.19) |  |
| *1/*17+*17/*17 | 10 | 0.31 (0.07-0.43) |  |
| ***CYP2D6*** |  |  |  |
| *1/*1 | 19 | 0.15 (0.10-0.21) | 0.228 |
| *1/*4 | 4 | 0.11 (0.08-0.16) |  |
| *4/*4 | 1 | 0.09 |  |
| ***CYP3A5*** |  |  |  |
| *1/*3 | 3 | 2.11 (1.85-2.76) | **0.001** |
| *3/*3 | 21 | 0.13 (0.07-0.18) |  |
| ***UGT1A1*** |  |  |  |
| *1/*1 | 8 | 1.51(1.31-2.07) |  |
| *1/*28 | 13 | 1.23(0.78-1.64) | 0.300 |
| *28/*28 | 3 | 0.34(0.31-1.53) |  |
| ***UGT2B7*** |  |  |  |
| *1/*1 | 6 | 1.36 (0.92-1.66) | 0.939 |
| *1/*2 | 15 | 1.52 (1.03-2.21) |  |
| *2/*2 | 3 | 1.24 (1.12-2.24) |  |
| ***UGT2B15a*** |  |  |  |
| *G/*G | 10 | 0.45 (0.24-0.51) | 0.072 |
| *G/*T | 8 | 0.59 (0.51-0.89) |  |
| *T/*T | 6 | 0.25 (0.13-0.40) |  |
| ***UGT2B15b*** |  |  |  |
| *G/*G | 10 | 0.56 (0.38-0.81) | 0.433 |
| *T/*G | 8 | 0.46 (0.28-0.50) |  |
| *T/*T | 6 | 0.32 (0.18-0.55) |  |

**Supplementary table 6.** Analysis of an association between common genetic variants and protein abundance of the studied drug-metabolizing enzymes.

|  | **n** | **Median (Q1-Q3)** | ***p*** |
| --- | --- | --- | --- |
| **CYP2B6** |  |  |  |
| *1/*1 | 9 | 87.70 (45.31-181.76) | 0.238 |
| *1/*6 | 15 | 46.46 (30.00-197.21) |  |
| **CYP2C8** |  |  |  |
| *1/*1 | 18 | 540.64 (288.05-750.16) | 0.289 |
| *1/*3 | 5 | 152.62 (101.21-226.57) |  |
| *3/*4 | 1 | 317.32 |  |
| **CYP2C9** |  |  |  |
| *1/*1 | 13 | 846.93 (401.56-2858.21) | 0.811 |
| *1/*2 | 5 | 1242.47 (630.81-1473.15) |  |
| *1/*3 | 5 | 656.09 (517.40-1125.18) |  |
| *2/*3 | 1 | 1181.21 |  |
| **CYP2C19** |  |  |  |
| *1/*1 | 10 | 119.84 (41.92-258.64) | **0.042** |
| *1/*2 | 4 | 0.00 (0.00-23.03) |  |
| *1/*17+*17/*17 | 10 | 63.11 (26.24-125.81) |  |
| **CYP2D6** |  |  |  |
| *1/*1 | 19 | 182.18 (135.40-217.73) | 0.075 |
| *1/*4 | 4 | 103.55 (87.86-146.08) |  |
| *4/*4 | 1 | 0.00 |  |
| **CYP3A5** |  |  |  |
| *1/*3 | 3 | 327.80 (290.54-679.89) | **0.001** |
| *3/*3 | 21 | 33.44 (21.04-47.37) |  |
| **UGT1A1** |  |  |  |
| *1/*1 | 8 | 869.82(716.43-1050.52) |  |
| *1/*28 | 13 | 666.78(483.11-1016.09) | 0.270 |
| *28/*28 | 3 | 251.59(222.49-669.90) |  |
| **UGT2B7** |  |  |  |
| *1/*1 | 6 | 1183.43 (646.45-2160.26) | 0.922 |
| *1/*2 | 15 | 1068.05 (705.00-1338.10) |  |
| *2/*2 | 3 | 1172.61 (603.05-1446.92) |  |
| **UGT2B15a** | |  |  |
| *G/*G | 10 | 425.57 (320.37-580.56) | 0.051 |
| *G/*T | 8 | 813.89 (474.02-1193.76) |  |
| *T/*T | 6 | 407.73 (288.39-520.03) |  |
| **UGT2B15b** | |  |  |
| *G/*G | 10 | 475.83 (234.13-791.37) | 0.448 |
| *T/*G | 8 | 407.73 (348.03-546.74) |  |
| *T/*T | 6 | 567.98 (432.64-836.41) |  |

**Supplementary table** 7. Linear regression analysis of relation between patient’s age and DME genes’ expression in human liver

|  | **protein** | | ***mRNA*** | |
| --- | --- | --- | --- | --- |
|  | **R^2^** | ***p*** | **R^2^** | ***p*** |
| ***CYP1A2*** | 0.002 | 0.846 | 0.002 | 0.832 |
| ***CYP2B6*** | 0.071 | 0.207 | 0.053 | 0.279 |
| ***CYP2C8*** | 0.048 | 0.306 | 0.004 | 0.766 |
| ***CYP2C9*** | 0.040 | 0.347 | 0.042 | 0.339 |
| ***CYP2C19*** | 0.000 | 0.926 | 0.013 | 0.589 |
| ***CYP2D6*** | 0.012 | 0.612 | 0.022 | 0.487 |
| ***CYP2E1*** | 0.049 | 0.298 | 0.005 | 0.755 |
| ***CYP3A4*** | 0.012 | 0.618 | 0.057 | 0.261 |
| ***CYP3A5*** | 0.058 | 0.258 | 0.050 | 0.291 |
| ***UGT1A1*** | 0.234 | **0.017** | 0.115 | 0.106 |
| ***UGT1A3*** | 0.100 | 0.132 | 0.127 | 0.088 |
| ***UGT2B7*** | 0.008 | 0.687 | 0.050 | 0.295 |
| ***UGT2B15*** | 0.149 | 0.063 | 0.081 | 0.178 |

**Supplementary table 8.** Analysis of mRNA relative quantity and protein abundance of DMEs in male and female subjects.

|  | **protein (fmol/mg)** |  | **mRNA** |  |
| --- | --- | --- | --- | --- |
|  | **Median (Q1-Q3)** | ***p*** | **Median (Q1-Q3)** | ***p*** |
| **CYP1A2** |  |  |  |  |
| F | 535.91 (358.48-875.49) | 0.834 | 0.28 (0.10-0.57) | 0.653 |
| M | 550.58 (297.84-1236.97) |  | 0.44 (0.13-0.66) |  |
| **CYP2B6** |  |  |  |  |
| F | 41.07 (30.55-70.99) | 0.238 | 0.86 (0.14-1.53) | 0.610 |
| M | 119.17 (37.79-211.51) |  | 0.99 (0.32-1.97) |  |
| **CYP2C8** |  |  |  |  |
| F | 305.46 (150.33-540.64) | 0.238 | 7.07 (4.29-8.83) | 0.172 |
| M | 489.15 (284.05-778.40) |  | 9.35 (5.61-13.43) |  |
| **CYP2C9** |  |  |  |  |
| F | 528.82 (290.37-1357.81) | 0.153 | 1.73 (1.05-2.01) | 0.153 |
| M | 1153.20 (556.18-2572.48) |  | 2.24 (1.48-2.61) |  |
| **CYP2C19** |  |  |  |  |
| F | 43.98 (23.56-154.73) | 0.787 | 0.27 (0.12-0.60) | 0.528 |
| M | 85.11 (12.87-147.70) |  | 0.31 (0.14-0.84) |  |
| **CYP2D6** |  |  |  |  |
| F | 164.76 (85.76-208.06) | 0.742 | 0.13 (0.09-0.21) | 0.881 |
| M | 179.65 (110.41-217.73) |  | 0.15 (0.10-0.20) |  |
| **CYP2E1** |  |  |  |  |
| F | 1354.49 (1186.06-2624.20) | 0.569 | 25.09 (19.11-34.89) | 0.569 |
| M | 1568.83 (1105.34-2314.71) |  | 22.91 (17.40-28.62) |  |
| **CYP3A4** |  |  |  |  |
| F | 410.45 (181.71-959.59) | 1.000 | 3.93 (0.66-5.70) | 0.834 |
| M | 595.29 (111.50-1038.34) |  | 2.78 (0.91-5.64) |  |
| **CYP3A5** |  |  |  |  |
| F | 37.68 (18.46-50.81) | 0.61 | 0.15 (0.07-0.19) | 0.653 |
| M | 40.65 (28.10-57.64) |  | 0.15 (0.10-0.24) |  |
| **UGT1A1** |  |  |  |  |
| F | 667.20 (281.41-1010.10) | 0.881 | 1.61 (1.15-1.84) | 0.610 |
| M | 723.24 (350.33-1052.39) |  | 1.24 (0.75-2.64) |  |
| **UGT1A3** |  |  |  |  |
| F | 144.39 (79.20-195.89) | 0.61 | 0.07 (0.05-0.14) | 0.120 |
| M | 136.72 (98.02-203.92) |  | 0.13 (0.09-0.20) |  |
| **UGT2B7** |  |  |  |  |
| F | 1035.84 (645.20-1392.51) | 0.697 | 1.55 (0.89-2.23) | 0.834 |
| M | 1069.15 (713.84-1807.11) |  | 1.48 (1.09-1.65) |  |
| **UGT2B15** |  |  |  |  |
| F | 435.01 (416.68-598.25) | 0.976 | 0.35 (0.19-0.64) | 0.452 |
| M | 516.48 (323.83-745.19) |  | 0.48 (0.35-0.61) |  |
